# Evaluation of Maxillary Dentoalveolar Expansion with Clear Aligners: A Retrospective CBCT Study

**DOI:** 10.3390/diagnostics15131586

**Published:** 2025-06-23

**Authors:** Monica Macrì, Silvia Medori, Felice Festa

**Affiliations:** Department of Innovative Technologies in Medicine & Dentistry, University “G. D’Annunzio” of Chieti–Pescara, 66100 Chieti, Italy

**Keywords:** 3D, CBCT, clear aligners, digital dentistry, maxillary expansion

## Abstract

**Background/Objectives:** Currently, clear aligners are preferred to conventional appliances, especially among adult patients. However, the use of aligners for treating maxillary constriction is still debated in the literature. Therefore, the purpose of this study was to assess maxillary dentoalveolar expansion following clear aligner therapy in adults using CBCT scans. **Methods**: The study sample encompassed 50 non-growing patients (27 females and 23 males) aged 20 to 42 undergoing clear aligner orthodontics without dental extractions or auxiliaries. Transverse linear distances were measured on initial and final CBCTs and, subsequently, analysed through paired *t*-test and ANOVA. We considered alveolar bone measurements and interdental widths measured at the buccal apices and cusps from canines to second molars. **Results**: The buccal alveolar ridge width showed the greatest expansion (1.01 ± 0.38 mm), followed by the palatal alveolar ridge and maxillary alveolar bone. Statistically significant improvements were observed for all interdental measurements. The most considerable changes occurred in the interpremolar cusp distances, while the least changes were seen in the intermolar apex distances. At the cusp level, the average interpremolar widths increased by 3.44 ± 0.22 mm for the first premolars and 3.14 ± 0.27 mm for the second ones. **Conclusions**: Clear aligner treatment can effectively manage a constricted maxillary arch. We found significant changes in the maxillary alveolar bone. Both inter-apex and inter-cusp widths increased in all teeth, with the highest values in the premolars. Moreover, the increases in interdental distances at both apex and cusp levels were related to tooth position.

## 1. Introduction

A constricted maxillary arch is one of the most common concerns in orthodontics [1,2]. On clinical examination, it is possible to find some peculiar signs of a maxillary deficiency, such as dental crowding, the presence of posterior crossbites, and dark buccal corridors [3].

An underdeveloped maxilla is strictly correlated with the evolutionary changes of the human species. The modern human face exhibits a different distribution of bone resorption and deposition areas compared to our predecessors [4]. The anterior portion of the maxilla has been subjected to continuous bone resorption phenomena during evolution; consequently, the maxillary length and width of modern faces are more reduced than those of ancient skulls [5].

The therapeutic approach of a transverse maxillary constriction can be a dentoalveolar or a skeletal expansion depending on the aetiology and the patient’s demographic and skeletal factors [6,7]. The choice of a conventional palatal separation is related to midpalatal suture maturation, which, in turn, is affected by age, sex, and skeletal growth patterns [8].

With the midpalatal suture closure, the possibility of a conventional expansion is reduced; therefore, the clinician generally opts for a dentoalveolar expansion in patients without skeletal discrepancies. Quad helix devices and conventional/self-ligating brackets or aligner appliances can be used for dentoalveolar expansion [9,10]. In fact, in cases of a constricted maxilla, a non-extraction therapy using multibracket appliances or removable aligners can increase the arch perimeter through the transverse expansion and/or proclination of anterior teeth: this non-extraction approach is particularly suitable for non-growing individuals [11,12].

In recent years, the demand for orthodontic therapies compatible with aesthetic and social needs has been increasing [13,14]. Clear aligners are a viable alternative to traditional orthodontic appliances; in fact, aligners provide several benefits, including better oral hygiene, greater comfort, fewer appointments, and improved aesthetics [15]. Thanks to the ongoing improvements in technology, materials, attachment designs and the introduction of virtual radicular setups, the clear aligners can even manage the most complex malocclusions [16,17]. Conversely, some clinicians emphasise the need for additional aligners or mid-course corrections to achieve the planned outcomes [18,19].

Most previous papers regarding clear aligners evaluated the effectiveness of dental movements, e.g., derotation, molar distalisation, intrusion, or extrusion, whereas a limited number of works have focused on the evaluation of a transverse maxillary increase [20,21].

The studies now available in the literature investigated the accuracy of maxillary expansion following clear aligners mainly by the analysis of pre- and post-treatment digital models or by the comparison of the final digital model with the virtual setup [22,23]. Moreover, some papers compared the arch expansion achieved by aligners with that attained by the multibracket technique [24,25]. Different primary features, i.e., materials, gingival margin designs, attachments, or auxiliaries, contributed to heterogeneous outcomes [23].

A recent review reported greater transverse inter-cusp widths after clear aligners, especially in the premolar area [26]. Additionally, a wider arch after orthodontics was related to the buccal tipping of crowns, which was more pronounced in anterior than posterior regions. Therefore, Bouchant et al. concluded that the expansion achieved by means of the aligners was predominantly dentoalveolar [26]. However, previous studies concerning maxillary expansion focused mainly on the coronal or gingival measurements, neglecting the analysis of root movements [27,28]. Furthermore, some previous papers were conducted on older materials or small study groups [27,29].

Cone-beam computed tomography (CBCT) provides an accurate evaluation of maxillary constriction, as anatomical measurements can be taken accurately [30]. Unlike 2D radiographic examinations, CBCT offers 3D images of distinct orofacial structures without the superimposition of contiguous anatomies [31]. Furthermore, using CBCT scans, clinicians can examine the entire tooth from the apex to the cusp thanks to multiple cuts. A few previous papers have three-dimensionally investigated the transverse expansion after clear aligner therapy using CBCTs [12,32]. Nevertheless, these works comprised a small sample size and focused on posterior areas. In a 2023 study, we noted the significant torque changes without substantial apical root resorption following expansion therapy using aligners; however, in that 3D analysis, we did not comprise the variations in the maxillary bone and interdental widths [33]. To date, a complete 3D analysis to assess dentoalveolar changes after non-extraction therapy with clear aligners has not yet been proposed in the literature.

Therefore, the current study aimed to evaluate the effects of clear aligners on the maxillary dentoalveolar expansion in adult patients using CBCT scans. Three null hypotheses were formulated.

The first null hypothesis was that there were no significant modifications in maxillary alveolar bone after clear aligner treatment.

The second null hypothesis was that there were no significant changes in inter-apex and inter-cusp widths from canines to second molars at the end of the therapy.

Lastly, the third null hypothesis was that there were no significant differences in pre- to post-treatment widths among tooth types.

## 2. Materials and Methods

We conducted a retrospective study involving patients who underwent orthodontic treatment with clear aligners between October 2021 and May 2024 in the Department of Innovative Technologies in Medicine & Dentistry at “G. d’Annunzio” University of Chieti-Pescara.

Ethics approval (number 23, 8 November 2018) was obtained by the Independent Ethics Committee of Chieti Hospital. The study protocol was drawn following the European Union Good Practice Rules and the Helsinki Declaration.

The patients were selected according to the inclusion and exclusion criteria. The inclusion criteria encompassed non-growing patients of both genders aged over 18 years; non-extraction treatment requiring a transverse dentoalveolar expansion of the maxillary arch exclusively with clear aligners in both arches; normodivergent subjects with a skeletal Class I malocclusion; the presence of all permanent teeth, except the wisdom teeth; and good collaboration throughout the entire duration of the treatment. The exclusion criteria were as follows: individuals with systemic diseases, orofacial abnormalities, trauma, and/or drug-taking history affecting tooth movement; extraction cases; subjects with molar crossbites; patients with temporomandibular disorders; the use of orthodontic auxiliaries during the arch expansion stage, such as cross elastics or mini-screws; orthognathic surgical cases; and previous orthodontic or orthopaedic treatments.

A total of 50 patients (27 females and 23 males) were eligible for this retrospective study. The participants’ ages ranged between 20 and 42 years, with an average age of 31.4 years. All patients provided written informed consent before the initiation of the orthodontic therapy. The therapeutic approach encompassed a maxillary dentoalveolar expansion exclusively with Invisalign^®^ clear aligners (Align Technology, Inc., Tempe, AZ, USA) in order to resolve any possible crowding and transverse discrepancy. The total number of aligners ranged from 29 to 40 per arch, with an average of 35 aligners. All participants were instructed to change the aligner every 14 days and to wear it for at least 22 h/day. The orthodontic treatment lasted, on average, 18 months.

During the initial visit (T0), the dental and medical histories, along with the orthodontic and gnathological clinical examinations, were collected for each participant. Additionally, a CBCT scan was prescribed for every participant.

CBCT scans were taken through NewTom VGi evo unit (NewTom–Cefla S.C., Imola, BO, Italy) according to the low dose protocol with these parameters: acquisition time of 15 s, 80 kVp, 5 mA, 35 microSievert (μSv), a field of view (FOV) of 240 × 190 mm, and normal image resolution [34]. During the CBCT procedure, all subjects were seated upright with their backs as perpendicular to the floor as possible, and their heads stabilised by ear rods in the external auditory meatus in order to orient the head according to the Natural Head Position (NHP). Each individual was advised to gaze into a mirror placed 1.5 m away. The NHP is regarded as a physiological and repeatable posture used for morphological analysis [35,36]. All patients were informed to swallow, bite down into centric occlusion with the lips in gentle contact, and refrain from moving.

**Figure 1:**
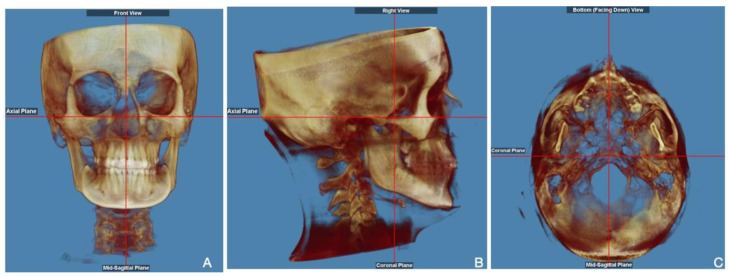
Three-dimensional skull orientation: front (**A**), right (**B**), and bottom (**C**) views in a similar way as Macrì et al. [33,37].

After X-ray scanning, DICOM (Digital Imaging and Communications in Medicine) image files were processed by Dolphin Imaging 3D software 11.9 (Dolphin Imaging & Management Solutions, Chatsworth, CA, USA) for a 3D analysis. To perform repeatable measurements, the patient’s skull image was oriented according to the NHP in the three planes of space orthogonal to each other, in a similar way as previous research (Figure 1): in the coronal orientation, the axial plane passing through the right Orbital (Or) is perpendicular to the mid-sagittal plane (MSP) passing through the Crista Galli (Cg); in the sagittal orientation, the Frankfurt plane (FH), a plane passing through two points, the right Orbital (Or) and Porion (Po), is perpendicular to the coronal plane; in the axial orientation, the coronal plane is perpendicular to the MSP passing through the midpalatal suture [33,37].

Consequently, the virtual 2D radiograms were extracted. The cephalometric analysis was performed on virtual lateral teleradiography to assess the sagittal and vertical skeletal patterns.

After acquiring intraoral and extraoral photos, the dental arches were scanned using an iTero Element 5D Plus^®^ scanner (Align Technology, Inc., Tempe, AZ, USA).

The virtual setup for each subject included a maxillary expansion and, except for the attachments, no auxiliaries were inserted. A single orthodontist with extensive knowledge of the clear aligner system planned the treatment using ClinCheck Pro^®^ 6.0 software (Align Technology, Inc., Tempe, AZ, USA). The fit of the aligners and the presence of required attachments were checked at each scheduled visit by the same orthodontist.

At the end of clear aligner therapy (T1), extraoral and intraoral photos and a CBCT scan were performed for each participant, and virtual 2D radiograms were acquired, as reported above. 

The coronal and the axial slices were examined to assess the maxillary expansion following clear aligner therapy.

All initial (T0) and final (T1) linear measurements were performed through the measurement tools present in Dolphin Imaging 3D software.

On the coronal slice, we examined the maxillary alveolar bone widths at T0 and T1, while on the axial slice, we analysed the inter-apex and inter-cusp widths in the maxillary canines, first and second premolars, and first and second molars at T0 and T1.

**Figure 2:**
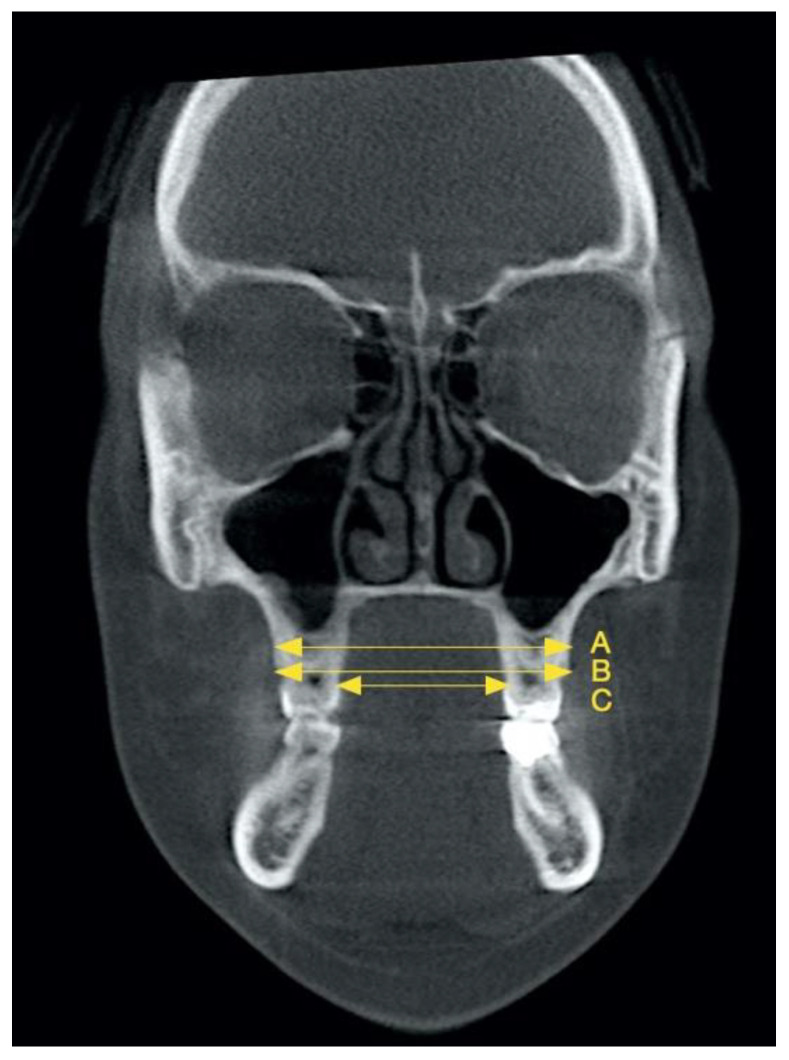
Maxillary linear distances measured in a similar way as Zhou and Guo: maxillary alveolar bone width (**A**); buccal alveolar ridge width (**B**); palatal alveolar ridge width (**C**).

Figure 2 reports the three distances at the first molar level taken on coronal slices in a similar way as Zhou and Gou [32]. We performed the following linear tomographic measurements regarding the maxillary alveolar bone: (A) the width at the level of the most convex points of the bilateral buccal alveolar bone; (B) the distance between the buccal alveolar ridges; and (C) the distance between the palatal alveolar ridges.

In addition, we performed transverse measurements on the superior submento-vertex.

**Figure 3:**
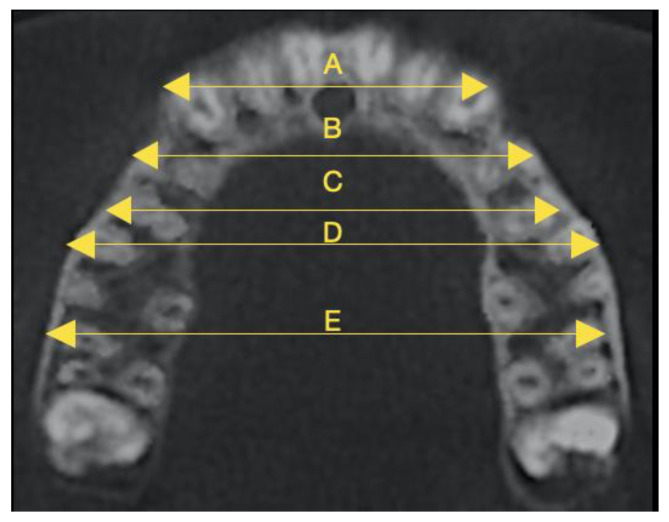
Distances of buccal root apices between the maxillary canines (**A**), first premolars (**B**), second premolars (**C**), first molars (**D**), and second molars (**E**).

The intercanine apex width was defined as the distance between the root apices of the right and left canines (Figure 3).

The first and second interpremolar apex widths were the distances from the buccal apices of the first and second premolars to their contralateral ones.

The first and second intermolar apex widths were described as the distances between the mesiobuccal apices of the first and second molars and their contralateral ones, in a similar way as Zhou and Guo [32].

**Figure 4:**
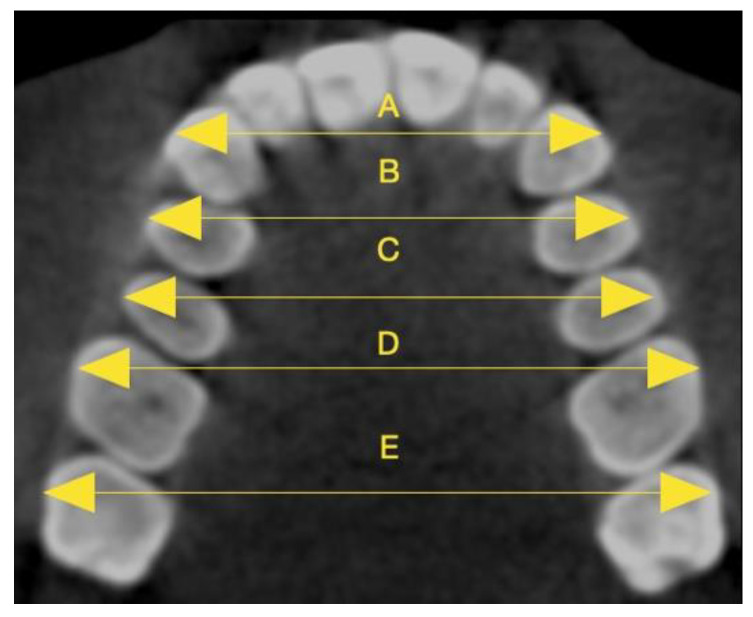
Linear measurements between the cusps of canines (**A**), the buccal cusps of first premolars (**B**) and second premolars (**C**), and the mesiobuccal cusps of first molars (**D**) and second molars (**E**).

The intercanine cusp width was defined as the distance between the cusps of the canines on each side of the maxilla (Figure 4).

The first and second interpremolar cusp widths were the distances from the buccal cusps of the first and second premolars to their contralateral ones.

The first and second intermolar cusp widths were described as the distances between the mesiobuccal cusps of the first and second molars and their contralateral ones, in a similar way as Morales-Burruezo et al. [38].

### Statistical Analysis

We estimated the size of the sample population in a similar way as Galluccio et al. [39]. Ultimately, we included 50 patients, providing 92% power for our observed effect (d = 0.62) and enhancing sensitivity for smaller effects. All linear measurements were conducted by a single trained examiner. After two weeks, the same investigator repeated all measurements on 20% of the study sample that was randomly selected. All distances previously exposed were assessed on a sample of ten patients as a calibration before the initiation of the study in a similar way as Perrotti et al. [10]. For each distance examined, the same measurement was repeated six times to potentially achieve the most accurate result, which was then indicated as the mean ± SD. The actual measurements were recorded for 50 participants only when the SD was below 5% of the average distance.

The intraclass correlation coefficient (ICC) was used to evaluate the intra-examiner measurement error. All initial and final measurements were recorded in a Microsoft Excel spreadsheet (version 2019; Microsoft, Redmond, WA, USA) to calculate means, standard deviations, and maximum and minimum values. A paired *t*-test was run to identify any significant variations in alveolar bone and interdental widths from the beginning to the end of the orthodontic therapy. A one-way ANOVA was performed to evaluate whether pre- to post-treatment width changes differed significantly among tooth types. The level of significance was set at 5%. All statistical analyses were executed using Stata 18 (version 18.0, 2023; StataCorp Llc., College Station, TX, USA).

## 3. Results

The current study analysed the transverse linear distances of the maxillary arch at T0 and T1 in 50 adult patients treated exclusively with clear aligners. All measurements were conducted on CBCT scans. ICC varied from 0.950 to 0.974, demonstrating high reliability.

Table 1 shows the modifications in maxillary alveolar bone widths following an orthodontic expansion. The greatest mean variation was detected in the buccal alveolar ridges (1.01 ± 0.38 mm). The average increase between the palatal alveolar ridges was 0.81 ± 0.37 mm. The increase in the maxillary width at the alveolar bone was less noticeable (0.09 ± 0.22 mm).

The paired *t*-test revealed statistically significant increases in both buccal and palatal alveolar ridges. We also observed a statistically significant increase in alveolar bone widths after clear aligner therapy (*p* < 0.01).

Concerning the changes in inter-apex widths, we found the highest mean expansion in the first premolars (1.89 ± 0.39 mm) while the lowest mean variation was found in the second molars (0.15 ± 0.09 mm). Similar mean values were observed in the canines and second premolars, 1.31 ± 0.73 mm and 1.48 ± 0.68 mm, respectively. A slight mean increase was noted in the first molars (0.70 ± 0.26 mm). For all teeth, the inter-apex distances were statistically significant (Table 2).

Table 3 illustrates the variations in inter-cusp distances from maxillary canines to second molars following an orthodontic expansion.

At the end of therapy, the greatest mean widths were found in the first premolars (3.44 ± 0.22 mm) followed by second premolars (3.14 ± 0.27 mm) and canines (2.32 ± 0.33 mm). The smallest mean widths occurred in the second molars (0.70 ± 0.28 mm). The average first intermolar cusp width was 2.60 ± 0.52 mm.

We found a statistically significant increase in inter-cusp widths from the canines to the second molars after clear aligner treatment.

The ANOVA analysis revealed a statistically significant main effect of tooth position (*F* (4, 245) = 95.36, *p* < 0.001), indicating substantial variation in treatment response regarding inter-apex widths across tooth types (Table 4). Post hoc comparisons using the Tukey’s Honestly Significant Difference (HSD) test demonstrated that the second molars exhibited significantly lower expansion (−1.74 ± 0.10 mm) compared to the first premolars at the apex level (*p* < 0.001). No significant inter-apex differences were detected between the second premolars (0.17 ± 0.10 mm) and canines (*p* = 0.405) (Table 5).

The changes in inter-cusp widths among teeth after clear aligner therapy were statistically significantly affected by tooth position (*F* (4, 245) = 487.29, *p* < 0.001) (Table 6). Regarding the inter-cusp widths, Tukey’s HSD test indicated that the second intermolar distances showed significantly lower increases (−2.74 ± 0.07 mm) compared to the first interpremolar ones (*p* < 0.001) (Table 7).

## 4. Discussion

The current study investigated the efficacy of the maxillary expansion after clear aligner therapy. Unlike other studies, we conducted a 3D analysis using CBCT scans. Moreover, we recruited only adults of both sexes across similar age groups to obtain a more homogeneous study sample. None of our participants showed a severe discrepancy between the two jaws, for which a combined surgical and orthodontic treatment could be necessary; consequently, a maxillary dentoalveolar expansion was planned to correct the maxillary arch morphology/constriction.

In the current study, we excluded children and adolescents. Indeed, residual growth could lead to further maxillary expansion, compromising the study outcomes. Furthermore, we did not encompass patients over 45 years, as the perimenopause period or any hormonal therapies could affect bone metabolism [40,41].

Moreover, we did not consider cases requiring crossbite elastics, mini-screws, or sectional wires. The use of these auxiliaries could facilitate maxillary arch expansion, reducing the therapy duration or achieving potentially greater expansion [42,43].

Our research revealed that clear aligner therapy can promote maxillary dentoalveolar expansion among adult patients with permanent dentition. Indeed, we found significant modifications in maxillary alveolar bone from the beginning to the end of the treatment, rejecting the first null hypothesis. We observed broader widths after clear aligner treatment, especially in the buccal and palatal alveolar ridges. To date, only one previous study examined alveolar bone distances [32]; however, with the exception of the buccal alveolar ridge, our results were consistent with those obtained by Zhou and Gou, who used the same software as we did [32].

We detected significant inter-apex and inter-cusp changes in each tooth type after clear aligner therapy, rejecting the second null hypothesis. Regarding the variation in inter-apex widths, we found statistically significant changes, with higher mean values for canines and premolars. The canines showed a lower increase than premolars, probably because the canines have a stronger and longer root and are positioned at the curvature of the maxillary arch; consequently, the canines may not receive adequate orthodontic force [44]. In the literature, only one previous study analysed the inter-apex distances; however, the authors described only the first intermolar apex widths with lower values than ours [32]. To date, the current analysis is the first research evaluating the inter-apex widths from canines to the second molars following a maxillary expansion treatment with clear aligners. Therefore, we lack a basis for comparison with previous studies. However, as reported in our previous study, clear aligners can improve both sagittal root positions and faciolingual inclinations without a substantial root length loss [33].

Regarding inter-cusp distances, the increase was more effective in the maxillary premolars. All increments were statistically significant; however, lower significant values were detected for the second molars. In detail, we observed an average intercanine width increase of 2.32 mm. In the literature, the mean increase in the intercanine diameter ranged from 1 mm to 2.20 mm [44,45]. In the current study, the first and second interpremolar distances grew by 3.44 mm and 3.14 mm, respectively. Most previous studies generally reported lower expansion in the same areas [39,46]. The first intermolar distance increased by 2.60 mm. This value was larger than that found by Zhou and Guo, who reported a mean value of 1.47 mm using CBCT scans [32]. Other studies using digital models obtained a mean amount of expansion similar to ours [38,44]. Conversely, Deregibus et al. found a notable increase in molar widths [27]. Bucur et al. also observed a greater expansion in the first intermolar distance (3.67 mm), likely because they combined aligners with an interceptive myofunctional device [45]. In our study, the second intermolar distances showed lower expansion (average 0.70 mm), likely due to the already proper positioning of these teeth in most patients from the initiation of the treatment [38]. We emphasised that only a few papers examined the second intermolar width and reported an average increase ranging from 0.45 mm to 0.70 mm [38,44].

In our study, both inter-apex and inter-cusp widths differed significantly among teeth, rejecting the third null hypothesis. Indeed, the premolars exhibited greater expansions at both apex and cusp levels; in particular, at the end of the therapy, the first interpremolar distances were significantly wider than the second intermolar ones. Therefore, the premolars represented an area of the maxillary arch particularly susceptible to expansion therapy with clear aligners. In fact, patients typically present an Omega-shaped arch with a collapse of interpremolar widths prior to orthodontic therapy. These results of interpremolar cusp distances corroborate those of previous papers [47,48]. D’Antò et al. also reported that the premolar widths expanded more than other teeth, probably because premolars are located in a straight area of the arch; however, those authors obtained lower expansion after the first set of aligners (2.42 mm and 2.17 mm in the first and second premolar distances, respectively) [46]. As reported in previous studies, we observed a larger expansion in the first premolars compared to the second premolars [39,46]. In contrast, only two studies noted higher second interpremolar distances than the first interpremolar ones [38,44]. However, the aforementioned studies referred only to inter-cusp measurements on digital models.

Moreover, in line with previous studies, we observed that the amount of expansion progressively reduced from premolars to molars [44,46]. This could be related to anatomical or biomechanical aspects. Indeed, more complex root anatomy, denser cortical bone, thicker soft tissue of the cheeks, and more elevated occlusal load may undermine a complete expansion in molar areas. Moreover, the efficiency of buccally directed force gradually diminishes from mesial to distal regions, and the flexibility of the material is less adequate in molar regions [12]. Therefore, the orthodontist might consider an overcorrection of posterior widths during virtual setups in order to limit midcourse corrections or refinement requests [49]. In addition, the planning of updated attachments in maxillary molars may contribute to posterior expansion [50]. The intermolar expansion is predictable up to 2 mm per quadrant, whereas, in cases of greater expansions, elastics or auxiliaries could be required [51]. Additionally, a considerable expansion of up to 3 mm per quadrant should be planned cautiously in order to prevent gingival recession, dehiscence, and fenestration [52].

As reported above, previous studies analysed the effectiveness of clear aligners in managing maxillary constriction among adults [26,47]. However, none of those papers evaluated the overall 3D transverse changes in maxillary arch widths. Moreover, a comparison with those papers was complicated since the studies differed in the material used, the study design, the sample size, and the number of additional aligners [22]. For instance, the studies differed in reference points, especially at the gingival level. Indeed, the measurement of the transverse gingival width should not be regarded as a reliable technique: during virtual setup, the software automatically removes the gum from the digital model and randomly repositions it without any specific criteria [23,38].

The present study was conducted using CBCTs. To date, merely one prior study utilised the 3D images [32]. Compared to the analysis proposed by Zhou and Gou [32], we included more dental measurements extending from canines to second molars in a larger sample size. We found a broader expansion in both buccal and palatal ridges as well as a larger first intermolar width.

In addition, it should be emphasised that several factors are involved in orthodontic treatment with aligners, thus leading to different findings among papers. Firstly, the dental movements during clear aligner therapy are related to biomechanical factors, such as the attachments and their design or the auxiliaries [53,54]. The use of elastics could assist or accelerate the correction of posterior crossbites [43]. Secondly, the practitioner’s experience influences the result of the treatment. In fact, an experienced clinician can plan an overcorrection of dental movements or choose more effective expansion features. In our study, a single orthodontist with extensive competence in clear aligners planned and validated the virtual setups, thus eliminating the variability associated with the setups performed by different clinicians [55]. Lastly, the effectiveness of orthodontic force depends on tooth size and dimension.

Moreover, our participants switched aligners every two weeks, which enhanced the precision of the intended orthodontic movements. Al-Nadawi et al. observed statistically more accurate movements in posterior teeth in 14-day protocols than in 7- or 10-day regimens [56]. Similarly, O’Connor et al. noted a greater clinical expression of maxillary buccal expansion following a 14-day change approach [57].

None of our patients underwent CBCT scans solely for the study’s purposes. Moreover, we selected a low-dose protocol to minimise radiogenic exposure. We used a CBCT with an effective dose of 35 μm allowing a more precise evaluation than 2D techniques [34]. Indeed, CBCT is useful for estimating tooth torque, root position relative to cortical plates, and alveolar bone width as well as for assessing skeletal growth patterns or upper airways [58,59]. Therefore, low-dose CBCT scans surpass the limitations of conventional X-rays, permitting better images without structural overlaps, a shorter scan time, and, consequently, lower radiation doses [60]. This decreased scan time can also reduce the potential artefacts caused by possible patient movements [61].

The present study exhibited some limitations. We considered aligners produced by a single manufacturer. Aligners made of diverse materials could display distinctive biomechanical properties and, consequently, different expansion rates [62]. We regarded only the buccal cusps; consequently, we did not examine any dental rotations. We overlooked potential differences between sexes or among ages. Particularly, gender could affect dental movements [63]. Moreover, we did not classify the patients according to the requested expansion amount or the complexity of the initial transverse malocclusion. Lastly, we did not assess the effect of transverse expansion in the sagittal direction.

Our 3D study demonstrated the effectiveness of the aligners in maxillary expansion. In fact, we provided a complete analysis of maxillary dentoalveolar expansion, obtaining larger transverse distances of both the alveolar bone and dental arch at the end of clear aligner therapy. Moreover, the extent of expansion achieved was significantly related to tooth position. The premolars showed the greatest transverse increases.

Future works, especially with a prospective design, could confirm or even improve our results thanks to advances and new features that will be developed in the coming years. Furthermore, future papers could encompass a follow-up period to better understand whether the outcomes achieved by clear aligners remain stable over time.

## 5. Conclusions

In the current 3D study, clear aligners were effective in promoting maxillary dentoalveolar expansion.

- We obtained a significant increase in maxillary alveolar bone width, particularly in the buccal and palatal alveolar ridges.
- We observed an efficient transverse expansion at both apex and cusp levels from canines to second molars.
- The inter-apex and inter-cusp widths achieved at the end of therapy differed significantly in relation to tooth position. The interpremolar widths exhibited the most notable increase and the most effective variation in treatment response.

Therefore, non-growing subjects with a proper bone base proportion who seek aesthetic orthodontic treatment may satisfactorily undergo a maxillary expansion with aligners. Orthodontists should regard clear aligners as a dependable approach to achieving maxillary dentoalveolar expansion, especially when detecting a collapse in premolar areas.

## Figures and Tables

**Table 1:** Variation in maxillary alveolar bone widths between T0 and T1.

Maxillary Linear Width	T1-T0 (mm) (Mean ± SD)	T1-T0 Minimum–Maximum Values (mm)	T1-T0 (%) (Mean ± SD)	T1-T0 Minimum–Maximum Values (%)	*p*-Value
Alveolar bone	0.09 ± 0.22	0–1.20	0.14 ± 0.39	0–2.04	0.009 *
Buccal alveolar ridge	1.01 ± 0.38	0.30–2.80	1.80 ± 0.69	0.53 ± 5.03	2.83857 × 10^−24^ **
Palatal alveolar ridge	0.81 ± 0.37	0–1.30	2.24 ± 1.04	0–4.05	2.08145 × 10^−20^ **

SD indicates standard deviation; * *p* < 0.01; ** *p* < 0.0001.

**Table 2:** Variation in inter-apex widths from canines to second molars between T0 and T1.

Inter-Apex Width	T1-T0 (mm) (Mean ± SD)	T1-T0 Minimum–Maximum Values (mm)	T1-T0 (%) (Mean ± SD)	T1-T0 Minimum–Maximum Values (%)	*p*-Value
Intercanine	1.31 ± 0.73	0.10–2.50	4.39 ± 2.43	0.34–8.39	5.84596 × 10^−17^ **
First interpremolar	1.89 ± 0.39	0.7–2.6	5.18 ± 1.11	1.93–7.22	1.07006 × 10^−35^ **
Second interpremolar	1.48 ± 0.68	0.10–2.60	3.53 ± 1.63	0.24–6.15	2.33339 × 10^−20^ **
First intermolar	0.70 ± 0.26	0.10–1.20	1.40 ± 0.53	0.20–2.41	4.20634 × 10^−24^ **
Second intermolar	0.15 ± 0.09	0–0.40	0.29 ± 0.17	0–0.77	2.28014 × 10^−16^ **

SD indicates standard deviation. ** *p* < 0.0001.

**Table 3:** Variation in inter-cusp widths from canines to second molars between T0 and T1.

Inter-Cusp Width	T1-T0 (mm) (Mean ± SD)	T1-T0 Minimum–Maximum Values (mm)	T1-T0 (%) (Mean ± SD)	T1-T0 Minimum–Maximum Values (%)	*p*-Value
Intercanine	2.32 ± 0.33	1.40–3.60	7.37 ± 1.11	4.18–11.76	2.39215 × 10^−43^ **
First interpremolar	3.44 ± 0.22	3.10–3.90	8.65 ± 0.60	7.71–10.13	1.1346 × 10^−60^ **
Second interpremolar	3.14 ± 0.27	2.70–3.60	6.94 ± 0.65	5.71–8.13	5.72304 × 10^−54^ **
First intermolar	2.60 ± 0.52	0.40–3.40	5.74 ± 1.19	0.90–7.64	1.4256 × 10^−35^ **
Second intermolar	0.70 ± 0.28	0.10–1.80	1.24 ± 0.51	0.18–3.24	1.2904 × 10^−22^ **

SD indicates standard deviation. ** *p* < 0.0001.

**Table 4:** One-way ANOVA for the variation of the inter-apex widths among tooth types.

Source	Sum of Squares	Degrees of Freedom	Mean Square	F	*p*-Value
Between groups	94.1761567	4	23.5440392	95.36	<0.001
Within groups	60.4889981	245	0.24689387		
Total	154.665155	249	0.6211452		

**Table 5:** Tukey’s HSD test for the inter-apex widths.

Inter-Apex Width	Contrast	Standard Error	*t* Test	*p*-Value > |t|	[95% Confidence Interval]	
Tooth type						
First premolar vs. canine	0.586	0.0993768	5.90	<0.001	0.3128924	0.8591075
Second premolar vs. canine	0.174	0.0993768	1.75	0.405	0.0991075	0.4471075
First molar vs. canine	0.61	0.0993768	6.14	<0.001	0.8831075	0.3368925
Second molar vs. canine	1.158	0.0993768	11.65	<0.001	1.431108	0.8848925
Second premolar vs. first premolar	0.412	0.0993768	4.15	<0.001	0.6851075	0.1388924
First molar vs. first premolar	1.196	0.0993768	12.03	<0.001	1.469108	0.9228924
Second molar vs. first premolar	1.744	0.0993768	17.55	<0.001	2.017108	1.470892
First molar vs. second premolar	0.784	0.0993768	7.89	<0.001	1.057108	0.5108924
Second molar vs. second premolar	1.332	0.0993768	13.40	<0.001	1.605108	1.058892
Second molar vs. first molar	0.548	0.0993768	5.51	<0.001	0.8211075	0.2748925

**Table 6:** One-way ANOVA for the variation of the inter-cusp widths among tooth types.

Source	Sum of Squares	Degrees of Freedom	Mean Square	F	*p*-Value
Between groups	227.96456	4	56.9911399	487.29	<0.001
Within groups	28.6542002	245	0.116955919		
Total	256.61876	249	1.03059743		

**Table 7:** Tukey’s HSD test for the inter-cusp widths.

Inter-Cusp Width	Contrast	Standard Error	*t* Test	*p*-Value > |t|	[95% Confidence Interval]	
Tooth type						
First premolar vs. canine	1.118	0.0683976	16.35	<0.001	0.9300295	1.30597
Second premolar vs. canine	0.82	0.0683976	11.99	<0.001	0.6320295	1.00797
First molar vs. canine	0.276	0.0683976	4.04	0.001	0.0880295	0.4639705
Second molar vs. canine	1.622	0.0683976	23.71	<0.001	1.80997	1.43403
Second premolar vs. first premolar	0.298	0.0683976	4.36	<0.001	0.4859705	0.1100295
First molar vs. first premolar	0.842	0.0683976	12.31	<0.001	1.02997	0.6540295
Second molar vs. first premolar	2.74	0.0683976	40.06	<0.001	2.92797	2.55203
First molar vs. second premolar	0.544	0.0683976	7.95	<0.001	0.7319705	0.3560295
Second molar vs. second premolar	2.442	0.0683976	35.70	<0.001	2.62997	2.25403
Second molar vs. first molar	1.898	0.0683976	27.75	<0.001	2.08597	1.71003

## Data Availability

The data presented in this study are available on request from the corresponding author. The data are not publicly available due to privacy. All data and figures were obtained from patients recruited for the present study.

## References

[1] McNamara J.A. (2000). Maxillary transverse deficiency. Am. J. Orthod. Dentofac. Orthop..

[2] Srivastava S.C., Mahida K., Agarwal C., Chavda R.M., Patel H.A. (2020). Longitudinal Stability of Rapid and Slow Maxillary Expansion: A Systematic Review. J. Contemp. Dent. Pract..

[3] Wei N., Wang C., Zhang Y., Wei Y., Hu W., Yang X., Chung K.H. (2022). The Influence of the Maxillary Posterior Region on Smile Aesthetics in a Chinese Cohort. Int. Dent. J..

[4] Lacruz R.S., Bermúdez de Castro J.M., Martinón-Torres M., O’Higgins P., Paine M.L., Carbonell E., Arsuaga J.L., Bromage T.G. (2013). Facial morphogenesis of the earliest europeans. PLoS ONE.

[5] Festa F., D’Anastasio R., Benazzi S., Macrì M. (2024). Three-Dimensional Analysis of the Maxillary Sinuses in Ancient Crania Dated to the V–VI Centuries BCE from Opi (Italy): Volumetric Measurements in Ancient Skulls from the Necropolis of Opi, Abruzzi, Italy. Diagnostics.

[6] Huynh T., Kennedy D.B., Joondeph D.R., Bollen A.M. (2009). Treatment response and stability of slow maxillary expansion using Haas, hyrax, and quad-helix appliances: A retrospective study. Am. J. Orthod. Dentofac. Orthop..

[7] Andrucioli M.C.D., Matsumoto M.A.N. (2020). Transverse maxillary deficiency: Treatment alternatives in face of early skeletal maturation. Dental Press J. Orthod..

[8] Festa F., Festa M., Medori S., Perrella G., Valentini P., Bolino G., Macrì M. (2024). Midpalatal Suture Maturation in Relation to Age, Sex, and Facial Skeletal Growth Patterns: A CBCT Study. Children.

[9] Revankar A.V., Bhat S.S., Rozario J.E. (2023). A comparison of the quadhelix and the nickel-titanium palatal expander in the treatment of narrow maxillary arches: A prospective clinical study. J. Orthod. Sci..

[10] Perrotti G., Carrafiello A., Rossi O., Karanxha L., Baccaglione G., Del Fabbro M. (2022). Clinical Use of Aligners Associated with Nuvola^®^ OP System for Transverse Maxillary Deficiency: A Retrospective Study on 100 Patients. Int. J. Environ. Res. Public Health.

[11] Robertson L., Kaur H., Fagundes N.C.F., Romanyk D., Major P., Flores Mir C. (2020). Effectiveness of clear aligner therapy for orthodontic treatment: A systematic review. Orthod. Craniofac. Res..

[12] Santucci V., Rossouw P.E., Michelogiannakis D., El-Baily T., Feng C. (2023). Assessment of Posterior Dentoalveolar Expansion with Invisalign in Adult Patients. Int. J. Environ. Res. Public Health.

[13] Weir T. (2017). Clear aligners in orthodontic treatment. Aust. Dent. J..

[14] Livas C., Pazhman F.S., Ilbeyli Z., Pandis N. (2023). Perceived esthetics and value of clear aligner therapy systems: A survey among dental school instructors and undergraduate students. Dent. Press J. Orthod..

[15] Pacheco-Pereira C., Brandelli J., Flores-Mir C. (2018). Patient satisfaction and quality of life changes after Invisalign treatment. Am. J. Orthod. Dentofac. Orthop..

[16] Bastidas-Castillo D.A., Ramirez-Naranjo P. (2024). Surgery first with clear aligners for a Class II patient: Case report and literature review. J. Stomatol. Oral Maxillofac. Surg..

[17] Nshimiyimana E., Ubuzima P., Mukeshimana C., Michelogiannakis D., Mbyayingabo D., Mugabo E., Gakunzi D., Ndanga E., Mazimpaka P., Habumugisha J. (2025). Skeletal and dental open bite treatment using clear aligners and orthodontic miniscrew-anchored fixed appliances in permanent dentition: A systematic review. J. World Fed. Orthod..

[18] Vaid N.R., Sabouni W., Wilmes B., Bichu Y.M., Thakkar D.P., Adel S.M. (2022). Customized adjuncts with clear aligner therapy: “The Golden Circle Model” explained!. J. World Fed. Orthod..

[19] Kravitz N.D., Dalloul B., Zaid Y.A., Shah C., Vaid N.R. (2023). What percentage of patients switch from Invisalign to braces? A retrospective study evaluating the conversion rate, number of refinement scans, and length of treatment. Am. J. Orthod. Dentofac. Orthop..

[20] Bilello G., Fazio M., Amato E., Crivello L., Galvano A., Currò G. (2022). Accuracy evaluation of orthodontic movements with aligners: A prospective observational study. Prog. Orthod..

[21] D’Antò V., Bucci R., De Simone V., Ghislanzoni L.H., Michelotti A., Rongo R. (2022). Evaluation of Tooth Movement Accuracy with Aligners: A Prospective Study. Materials.

[22] Rocha A.S., Gonçalves M., Oliveira A.C., Azevedo R.M.S., Pinho T. (2023). Efficiency and Predictability of Coronal Maxillary Expansion Repercussion with the Aligners System: A Retrospective Study. Dent. J..

[23] Aragon M.L.S.C., Mendes Ribeiro S.M., Fernandes Fagundes N.C., Normando D. (2024). Effectiveness of dental arch expansion in the orthodontic treatment with clear aligners: A scoping review. Eur. J. Orthod..

[24] Gu J., Tang J.S., Skulski B., Fields H.W., Beck F.M., Firestone A.R., Kim D.G., Deguchi T. (2017). Evaluation of Invisalign treatment effectiveness and efficiency compared with conventional fixed appliances using the Peer Assessment Rating index. Am. J. Orthod. Dentofac. Orthop..

[25] Ke Y., Zhu Y., Zhu M. (2019). A comparison of treatment effectiveness between clear aligner and fixed appliance therapies. BMC Oral Health.

[26] Bouchant M., Saade A., El Helou M. (2023). Is maxillary arch expansion with Invisalign^®^ efficient and predictable? A systematic review. Int. Orthod..

[27] Deregibus A., Tallone L., Rossini G., Parrini S., Piancino M., Castroflorio T. (2020). Morphometric analysis of dental arch form changes in class II patients treated with clear aligners. J. Orofac. Orthop..

[28] Vidal-Bernárdez M.L., Vilches-Arenas Á., Sonnemberg B., Solano-Reina E., Solano-Mendoza B. (2021). Efficacy and predictability of maxillary and mandibular expansion with the Invisalign^®^ system. J. Clin. Exp. Dent..

[29] Solano-Mendoza B., Sonnemberg B., Solano-Reina E., Iglesias-Linares A. (2017). How effective is the Invisalign^®^ system in expansion movement with Ex30′ aligners?. Clin. Oral Investig..

[30] Abdelkarim A. (2019). Cone-Beam Computed Tomography in Orthodontics. Dent. J..

[31] Tang H., Liu S., Shi Y., Wei J., Peng J., Feng H. (2025). Automatic segmentation and landmark detection of 3D CBCT images using semi supervised learning for assisting orthognathic surgery planning. Sci. Rep..

[32] Zhou N., Guo J. (2020). Efficiency of upper arch expansion with the Invisalign system. Angle Orthod..

[33] Macrì M., Medori S., Varvara G., Festa F. (2023). A Digital 3D Retrospective Study Evaluating the Efficacy of Root Control during Orthodontic Treatment with Clear Aligners. Appl. Sci..

[34] Feragalli B., Rampado O., Abate C., Macrì M., Festa F., Stromei F., Caputi S., Guglielmi G. (2017). Cone beam computed tomography for dental and maxillofacial imaging: Technique improvement and low-dose protocols. Radiol. Med..

[35] Verma S.K., Maheshwari S., Gautam S.N., Prabhat K., Kumar S. (2012). Natural head position: Key position for radiographic and photographic analysis and research of craniofacial complex. J. Oral Biol. Craniofac. Res..

[36] Tian K., Li Q., Wang X., Liu X., Wang X., Li Z. (2015). Reproducibility of natural head position in normal Chinese people. Am. J. Orthod. Dentofac. Orthop..

[37] Macrì M., Toniato E., Murmura G., Varvara G., Festa F. (2022). Midpalatal Suture Density as a Function of Sex and Growth-Pattern-Related Variability via CBCT Evaluations of 392 Adolescents Treated with a Rapid Maxillary Expander Appliance. Appl. Sci..

[38] Morales-Burruezo I., Gandía-Franco J.L., Cobo J., Vela-Hernández A., Bellot-Arcís C. (2020). Arch expansion with the Invisalign system: Efficacy and predictability. PLoS ONE.

[39] Galluccio G., De Stefano A.A., Horodynski M., Impellizzeri A., Guarnieri R., Barbato E., Di Carlo S., De Angelis F. (2023). Efficacy and Accuracy of Maxillary Arch Expansion with Clear Aligner Treatment. Int. J. Environ. Res. Public Health.

[40] Deng L., Guo Y. (2020). Estrogen effects on orthodontic tooth movement and orthodontically-induced root resorption. Arch. Oral Biol..

[41] Krishnan V., Kuijpers-Jagtman A.M., Davidovitch Z. (2022). Biological Mechanisms of Tooth Movement.

[42] Rossini G., Parrini S., Castroflorio T., Deregibus A., Debernardi C.L. (2015). Efficacy of clear aligners in controlling orthodontic tooth movement: A systematic review. Angle Orthod..

[43] Houle J.P., Piedade L., Todescan R., Pinheiro F.H. (2017). The predictability of transverse changes with Invisalign. Angle Orthod..

[44] Lione R., Paoloni V., Bartolommei L., Gazzani F., Meuli S., Pavoni C., Cozza P. (2021). Maxillary arch development with Invisalign system. Angle Orthod..

[45] Bucur S.M., Moga R.A., Olteanu C.D., Bud E.S., Vlasa A. (2025). A Retrospective Study Regarding the Efficacy of Nuvola^®^ OP Clear Aligners in Maxillary Arch Expansion in Adult Patients. Diagnostics.

[46] D’Antò V., Valletta R., Di Mauro L., Riccitiello F., Kirlis R., Rongo R. (2023). The Predictability of Transverse Changes in Patients Treated with Clear Aligners. Materials.

[47] Ma S., Wang Y. (2023). Clinical outcomes of arch expansion with Invisalign: A systematic review. BMC Oral Health.

[48] Kalekar A.A., Manchanda J., Chavan S., Bhad W.A., Atram H., Badu P., Tarde P. (2024). Effectiveness of maxillary arch expansion using clear aligners in adult patients: A systematic review and meta-analysis. Aust. Orthod. J..

[49] Riede U., Wai S., Neururer S., Reistenhofer B., Riede G., Besser K., Crismani A. (2021). Maxillary expansion or contraction and occlusal contact adjustment: Effectiveness of current aligner treatment. Clin. Oral Investig..

[50] Papadimitriou A., Mousoulea S., Gkantidis N., Kloukos D. (2018). Clinical effectiveness of Invisalign^®^ orthodontic treatment: A systematic review. Prog. Orthod..

[51] Charalampakis O., Iliadi A., Ueno H., Oliver D.R., Kim K.B. (2018). Accuracy of clear aligners: A retrospective study of patients who needed refinement. Am. J. Orthod. Dentofac. Orthop..

[52] Ali S.A., Miethke H.R. (2012). Invisalign, an innovative invisible orthodontic appliance to correct malocclusions: Advantages and limitations. Dent. Update.

[53] Castroflorio T., Sedran A., Parrini S., Garino F., Reverdito M., Capuozzo R., Mutinelli S., Grybauskas S., Vaitiekūnas M., Deregibus A. (2023). Predictability of orthodontic tooth movement with aligners: Effect of treatment design. Prog. Orthod..

[54] Nucera R., Dolci C., Bellocchio A.M., Costa S., Barbera S., Rustico L., Farronato M., Militi A., Portelli M. (2022). Effects of Composite Attachments on Orthodontic Clear Aligners Therapy: A Systematic Review. Materials.

[55] Nogal-Coloma A., Yeste-Ojeda F., Rivero-Lesmes J.C., Martin C. (2023). Predictability of Maxillary Dentoalveolar Expansion Using Clear Aligners in Different Types of Crossbites. Appl. Sci..

[56] Al-Nadawi M., Kravitz N.D., Hansa I., Makki L., Ferguson D.J., Vaid N.R. (2021). Effect of clear aligner wear protocol on the efficacy of tooth movement. Angle Orthod..

[57] O’Connor J., Weir T., Freer E., Kerr B. (2024). Clinical expression of programmed maxillary buccal expansion and buccolingual crown inclination with Invisalign EX30 and SmartTrack aligners and the effect of 1-week vs. 2-week aligner change regimes: A retrospective cohort study. Korean J. Orthod..

[58] Dudic A., Giannopoulou C., Leuzinger M., Kiliaridis S. (2009). Detection of apical root resorption after orthodontic treatment by using panoramic radiography and cone-beam computed tomography of super-high resolution. Am. J. Orthod. Dentofac. Orthop..

[59] Deng Y., Sun Y., Xu T. (2018). Evaluation of root resorption after comprehensive orthodontic treatment using cone beam computed tomography (CBCT): A meta-analysis. BMC Oral Health.

[60] Mallya S.M., Lam E.W.N. (2018). White and Pharoah’s Oral Radiology.

[61] Venkatesh E., Elluru S.V. (2017). Cone beam computed tomography: Basics and applications in dentistry. J. Istanb. Univ. Fac. Dent..

[62] Lombardo L., Palone M., Longo M., Arveda N., Nacucchi M., De Pascalis F., Spedicato G.A., Siciliani G. (2020). MicroCT X-Ray Comparison of Aligner Gap and Thickness of Six Brands of Aligners: An in-Vitro Study. Prog. Orthod..

[63] Chisari J.R., McGorray S.P., Nair M., Wheeler T.T. (2014). Variables affecting orthodontic tooth movement with clear aligners. Am. J. Orthod. Dentofac. Orthop..

